# Modulation of microRNA-mRNA Target Pairs by Human Papillomavirus 16 Oncoproteins

**DOI:** 10.1128/mBio.02170-16

**Published:** 2017-01-03

**Authors:** Mallory E. Harden, Nripesh Prasad, Anthony Griffiths, Karl Munger

**Affiliations:** a Program in Virology, Division of Medical Sciences, Harvard Medical School, Boston, Massachusetts, USA; b Department of Developmental, Molecular and Chemical Biology, Tufts University School of Medicine, Boston, Massachusetts, USA; c Genomic Services Laboratory, HudsonAlpha Institute for Biotechnology, Huntsville, Alabama, USA; d Department of Virology and Immunology, Texas Biomedical Research Institute, San Antonio, Texas, USA; Icahn School of Medicine at Mount Sinai

## Abstract

The E6 and E7 proteins are the major oncogenic drivers encoded by high-risk human papillomaviruses (HPVs). While many aspects of the transforming activities of these proteins have been extensively studied, there are fewer studies that have investigated how HPV E6/E7 expression affects the expression of cellular noncoding RNAs. The goal of our study was to investigate HPV16 E6/E7 modulation of cellular microRNA (miR) levels and to determine the potential consequences for cellular gene expression. We performed deep sequencing of small and large cellular RNAs in primary undifferentiated cultures of human foreskin keratinocytes (HFKs) with stable expression of HPV16 E6/E7 or a control vector. After integration of the two data sets, we identified 51 differentially expressed cellular miRs associated with the modulation of 1,456 potential target mRNAs in HPV16 E6/E7-expressing HFKs. We discovered that the degree of differential miR expression in HFKs expressing HPV16 E6/E7 was not necessarily predictive of the number of corresponding mRNA targets or the potential impact on gene expression. Additional analyses of the identified miR-mRNA pairs suggest modulation of specific biological activities and biochemical pathways. Overall, our study supports the model that perturbation of cellular miR expression by HPV16 E6/E7 importantly contributes to the rewiring of cellular regulatory circuits by the high-risk HPV E6 and E7 proteins that contribute to oncogenic transformation.

## IMPORTANCE

High-risk human papillomaviruses (HPVs) are the causative agents of almost all cervical cancers and many other cancers, including anal, vaginal, vulvar, penile, and oropharyngeal cancers. Despite the availability of efficacious HPV vaccines, it is critical to determine how HPVs cause cancer, as many people remain unvaccinated and the vaccine does not prevent cancer development in individuals who are already infected. Two HPV proteins, E6 and E7, are the major drivers of cancer development, and much remains to be learned about how the expression of these viral proteins reprograms infected cells, ultimately resulting in cancer development. Small, noncoding human RNAs, termed microRNAs (miRs), regulate gene expression and have been implicated in almost all human cancers, including HPV-associated cancers. Our study provides a comprehensive analysis of how E6 and E7 alter the expression of human miRs and how this potentially impacts cellular gene expression, which may contribute to cancer development.

## INTRODUCTION

Human papillomaviruses (HPVs) are small, double-stranded DNA viruses that infect undifferentiated basal epithelial cells of stratified epithelia (reviewed in reference 1). A subset of HPVs classified as “high-risk” are the causative agents of almost all cervical cancers, as well as many other anogenital tract and oral carcinomas. The E6 and E7 proteins are consistently expressed in high-risk HPV^+^ lesions and cancers and are the main drivers of cell transformation (reviewed in references 2 and 3). HPV E6 and E7 are small proteins with no intrinsic enzymatic or DNA-binding activities that function by targeting host pathways that modulate multiple downstream effectors (reviewed in reference 1), thereby causing alterations in critical physiological processes deemed “hallmarks of cancer” (3, 4). Most notably, high-risk HPV E6 and E7 proteins bind and target the TP53 and retinoblastoma tumor suppressor protein RB1 (5) for proteasomal degradation (6–8). In addition, high-risk HPV E6 and E7 also interact with many other multifunctional, nonredundant proteins, including transcription factors and epigenetic regulators that, in turn, cause alterations in cellular gene expression. In addition to coding genes, high-risk HPV E6 and E7 also cause alterations in the expression of noncoding RNAs, including microRNAs (miRs) (9).

miRs are small (~22-nucleotide [nt]) noncoding RNAs that regulate target mRNAs at the posttranscriptional level. Most mammalian mRNAs are miR targets (10). Targeting involves binding of the miR seed (nt 2 to 7) to complementary sequences in target mRNAs, with most miR target sites mapping to 3′ untranslated regions (11). Regulation of target mRNAs can occur via mRNA destabilization, translational repression, or a combination of both mechanisms. Specifically, mRNA destabilization accounts for the majority of miR-mediated repression (12, 13), while only 10 to 25% of overall miR repression is due to inhibition of translation (14). Each individual miR can alter the expression of hundreds of targets (15), and mRNAs can be regulated by multiple miRs. Typically, miRs impart modest effects on any single target and are thought to balance or “fine-tune” gene expression. However, the additive effect of multiple miRs targeting a particular pathway or one miR targeting several components of a specific pathway can result in substantial biological consequences. Therefore, through manipulation of host miRs, HPV E6 and E7 may modulate many downstream mRNA targets involved in various biological processes.

At least one HPV type, HPV31, does not encode miRs (16). However, the possibility cannot be ruled out that some other HPVs may encode miRs. Regardless, by altering host miR expression, HPVs can reap many of the benefits achieved through viral miRs without encoding their own. To date, only two studies (17, 18) have used small RNA sequencing (miR-seq) to investigate alterations in host miRs in the context of high-risk HPV infection and these studies used organotypic raft cultures composed of epithelial cells undergoing differentiation. Given that HPVs, particularly the HPV E6 and E7 proteins, can alter epithelial cell differentiation and/or sustain cellular proliferation in differentiated cells (19), it is unclear whether the reported changes in miR levels are directly caused by HPV gene expression or whether they represent the consequence of HPV-induced changes in epithelial cell proliferation and differentiation.

To circumvent this complication, we aimed to investigate how the expression of high-risk HPV E6 and E7 modulates miR levels in homogeneous populations of undifferentiated primary human foreskin keratinocytes (HFKs). We performed deep sequencing of miRs from HFK populations with stable, low-level HPV16 E6/E7 expression and donor- and passage-matched control vector-transduced HFKs. To comprehensively capture the potential impact of miR regulation on cellular mRNA abundance, we performed deep sequencing of cellular RNAs (RNA-seq) that were simultaneously isolated from the identical HFK populations used for miR profiling. After pairing the miR expression data with the RNA expression data, we identified miRs that are likely to be functionally important in HPV16 E6/E7-expressing HFKs. Additional bioinformatic analyses revealed key canonical pathways that are specifically enriched in the identified miR-mRNA target pairs in comparison to the entire RNA-seq data set. Taken together, the results of our study show that modulation of cellular miR expression plays a substantial role in the HPV16 E6/E7-mediated reprogramming of cellular gene expression and may contribute importantly to the oncogenic activities of these proteins.

## RESULTS

### Expression of HPV16 E6/E7 in HFKs alters host miR expression profiles.

Alterations in miR levels in response to high-risk HPV16 E6/E7 expression in undifferentiated human epithelial cells has not been extensively studied. We used miR-seq to investigate the modulation of miR expression in two independent, donor- and passage-matched HFK populations, each with stable expression of HPV16 E6/E7 or a control vector. For the purpose of this analysis, we applied threshold cutoffs of ≥10 miR reads, a ±3.0-fold or greater change in expression, and a false-discovery rate (FDR) of ≤0.05. The results from the two control samples and the two samples with expression of HPV16 E6/E7 were averaged, and only miRs with consistent changes in both samples were considered in downstream analyses.

**FIG 1:**
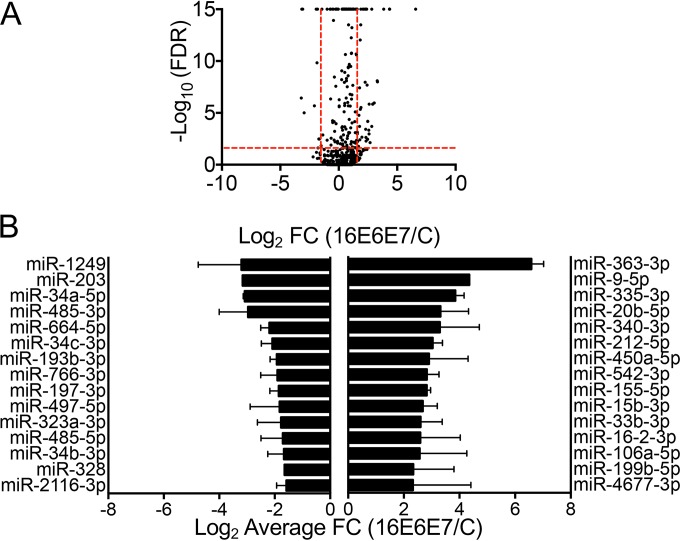
miR-seq of HPV16 E6/E7-expressing or control vector-transduced HFKs. (A) Volcano plot of miR-seq data with black dots representing the average expression of individual miRNAs in two HFK populations. The –log_10_-transformed FDR is plotted on the *y* axis, and the log_2_-transformed fold change (FC) in HPV16 E6/E7-expressing HFKs compared to controls is on the *x* axis. Vertical red lines indicate the FC thresholds (−3 ≥ FC ≥3), and horizontal red lines indicate the FDR threshold (FDR, ≤0.05). (B) Graph of the top 15 up- and downregulated miRs in HPV16 E6/E7-expressing HFKs. Error bars indicate the standard error of the mean.

**FIG 2:**
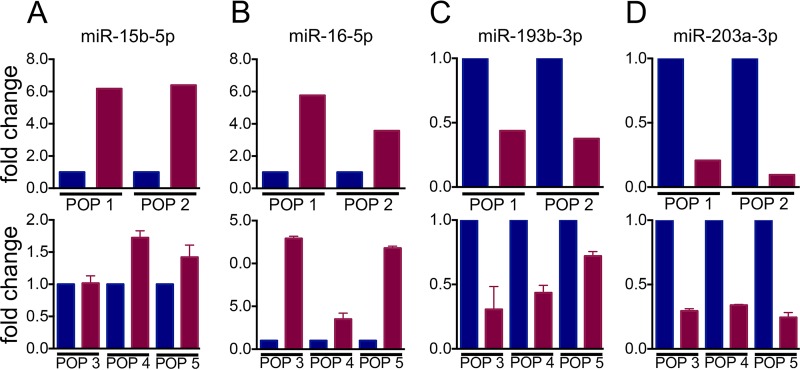
Validation of selected miRs identified by miR-seq. (A to D) Validation of miR-15b-5p (A), miR-16-5p (B), miR-193b-3p (C), and miR-203a-3p (D) levels in HPV16 E6/E7-expressing (red) and control vector-transduced (blue) HFKs. The top graphs show miR expression from two HFK populations determined by miR-seq. The bottom graphs show expression of the corresponding miRs in three additional HFK populations determined via RT-qPCR. Expression of the noncoding snRNA U6 spliceosomal RNA was used as an internal control in TaqMan miR assays. Results represent averages and standard deviations of at least three independent experiments.

A total of 2,104 (81%) of the 2,588 human miRs compiled in miRBase release 21 (20–24) were detected. By applying the threshold cutoffs, 78 miRs were differentially expressed in HPV16 E6/E7-expressing HFKs compared to the control vector-transduced populations (Fig. 1A). Of the 78 differentially expressed miRs, 62 were upregulated and 16 were downregulated. The top 15 most up- and downregulated miRs are shown in Fig. 1B. Additionally, while the most suppressed miR (miR-1249) was decreased 9.2-fold, five miRs were upregulated more than 9.2-fold. The expression of several miRs was confirmed via TaqMan miR assay in multiple additional HFK populations (Fig. 2A to D).

To understand the contributions of the individual oncoproteins to changes in miR expression, we investigated the expression of miRs in two matched HFK populations with expression of HPV16 E6 or HPV16 E7 alone. Table S1 in the supplemental material lists the top miRs that were consistently up- or downregulated in both HFK populations. Six miRs are upregulated by both HPV16 E6 and E7 (Table S1A and B). Similarly, five miRs are downregulated by both HPV16 E6 and E7 (Table S1C and D). Several miRs, miR-33b-3p, -542-3p, and -335-3p, are upregulated in HFKs expressing both HPV16 E6 and E7, as well as in HFKs expressing HPV16 E6 or E7 alone. Similarly, miR-193b-3p is downregulated in HPV16 E6/E7-expressing HFKs, as well as in HFKs expressing HPV16 E6 or E7 alone. However, the expression of some HPV16 E6/E7-modulated miRs is driven by one specific oncoprotein. Upregulation of miR-16-2-3p in HPV16 E6/E7-expressing HFKs is driven by E7 expression (Table S1A), whereas upregulation of miR-363-3p, -9-5p, and -450a-5p is driven by E6 (Table S1B). Downregulation of miR-197-3p and -1249 in HPV16 E6/E7-expressing HFKs is driven by E7 (Table S1C), whereas downregulation of miR-34a-5p and -34c-3p is driven by E6 (Table S1D). Hence, some miRs altered in HPV16 E6/E7-expressing HFKs are driven by the expression of both HPV16 E6 and E7, whereas others are independently modulated by E6 or E7 alone.

10.1128/mBio.02170-16.1 TABLE S1  miRs modulated in HFKs with expression of individual viral oncoproteins. Download Table S1, DOCX file, 0.1 MB. Copyright © 2017 Harden et al. 2017 Harden et al. This content is distributed under the terms of the Creative Commons Attribution 4.0 International license.

### Expression of HPV16 E6/E7 in HFKs alters human miR clusters.

Approximately 20% of all known human miRs are genomically clustered. On the basis of the miRBase (20–24) definition of a miR cluster, there are 153 genomic clusters made up of 465 human miRs (25). Many miR clusters have been shown to be coexpressed from the same primary miR transcript (25–28). Since the evolution of miR clusters is thought to have involved gene duplications, clustered miRs are often members of the same seed family (29–33). Of functional importance, altering expression of multiple miRs in a gene cluster may result in coordinated regulation of multiple biological processes (34).

Therefore, we assessed whether some of the differentially expressed miRs that met our threshold cutoffs were part of miR clusters. Additionally, for any HPV16 E6/E7-regulated miR associated with a cluster, we reexamined the expression of other miRs belonging to that cluster. This analysis showed that 35 of the 78 differentially expressed miRs were members of a genomic cluster and of these 35 miRs, 13 were found to be part of larger clusters (≥3 miRs), whereas the rest were members of small clusters containing only 2 miRs. Seven miRs, including miR-362, -106a, -20b, -363, -542, -450a-1, and -450a-2, were part of a cluster of ≥6 miRs, and miR-485 and -323a were part of a cluster of ≥13 miRs. As shown in Table S2, in some clusters, all of the miRs within the cluster show the same trend in expression. However, in other clusters, miRs within the cluster show a mixed trend in expression, with the expression of some miRs upregulated and that of some downregulated as a result of HPV16 E6/E7. In total, the expression of 26 miR clusters was altered in response to HPV16 E6/E7 expression, suggesting that HPV16 E6/E7 expression modulates individual miRs and miR clusters.

10.1128/mBio.02170-16.2 TABLE S2  miR clusters modulated by expression of HPV16 E6/E7. Download Table S2, DOCX file, 0.1 MB. Copyright © 2017 Harden et al. 2017 Harden et al. This content is distributed under the terms of the Creative Commons Attribution 4.0 International license.

### RNA-seq analysis of HPV16 E6/E7-expressing HFKs.

**FIG 3:**
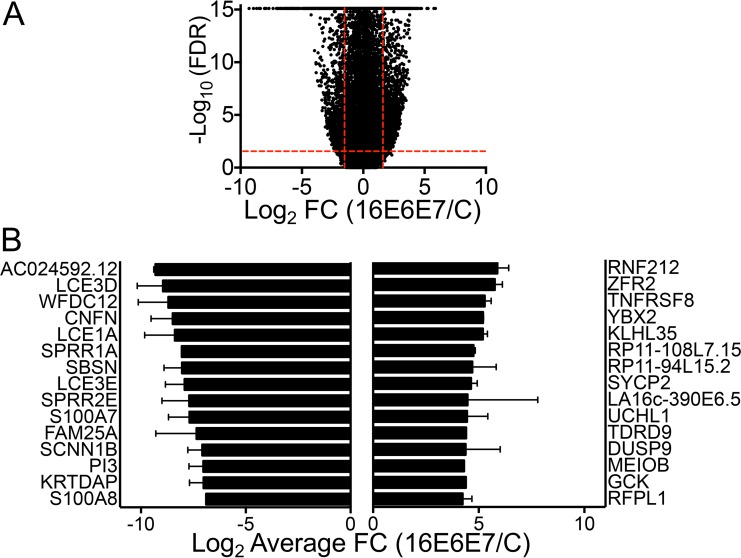
RNA-seq of large RNAs from HPV16 E6/E7-expressing or control vector-transduced HFKs. (A) Volcano plot of RNA-seq data with black dots representing the average expression of individual RNAs in two HFK populations. The –log_10_-transformed FDR is plotted on the *y* axis, and the log_2_-transformed fold change (FC) in HPV16 E6/E7-expressing HFKs compared to controls is on the *x* axis. Vertical red lines indicate the FC thresholds (−2 ≥ FC ≥2), and horizontal red lines indicate the FDR threshold (FDR, ≤0.05). (B) Graph of the top 15 up- and downregulated RNAs in HPV16 E6/E7-expressing HFKs. Error bars indicate the standard error of the mean.

To comprehensively assess the effects of the observed miR expression changes on potential target RNAs, we also performed RNA-seq with large RNAs (≥200 nt) that were simultaneously isolated from the same two independent populations of HFKs from which miR expression was analyzed. Similar to the miR-seq data, threshold cutoffs of ≥10 reads, a ±2.0-fold or greater change in expression, and an FDR of ≤0.05 were used for analysis of the RNA-seq data. A volcano plot of the RNA-seq data is shown in Fig. 3A. In total, 3,471 protein-coding RNAs, corresponding to 16% of all human protein-coding RNAs and 8.7% of Human Genome Organisation Gene Nomenclature Committee-approved genes (which include non-protein-coding genes) were significantly altered in HPV16 E6/E7-expressing HFKs (Table S3). More RNAs were downregulated than upregulated, and Fig. 3B shows the top 15 most up- and downregulated RNAs.

10.1128/mBio.02170-16.3 TABLE S3  Numbers and types of RNAs detected by RNA-seq. Download Table S3, DOCX file, 0.1 MB. Copyright © 2017 Harden et al. 2017 Harden et al. This content is distributed under the terms of the Creative Commons Attribution 4.0 International license.

High-risk HPV E6 proteins are known to increase the expression of the catalytic protein subunit of human telomerase, TERT (35), and TERT was upregulated in our HPV16 E6/E7-expressing HFKs. Similarly the high-risk HPV biomarker CDKN2A (p16^INK4A^) was expressed at higher levels in HPV16 E6/E7-expressing HFKs than in control HFKs. Consistent with TP53 inactivation by HPV16 E6 (36), lower levels of TP53 transcriptional targets, including CDKN1A (p21^CIP1^), BAX, GADD45A, and MDM2, were detected in HPV16 E6/E7-expressing HFKs than in controls. These observations suggest that our data agree with previously observed HPV-associated gene expression changes.

### Integration of miR-seq and RNA-seq data to identify potential miR-mRNA target pairs.

**FIG 4:**
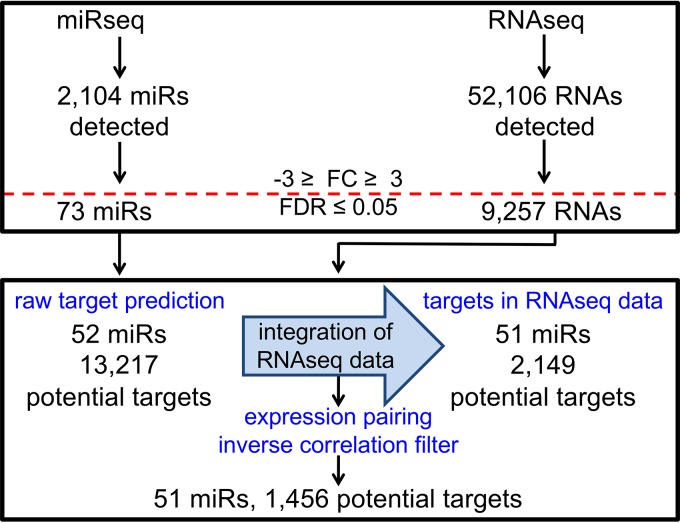
Schematic of the miR-mRNA expression-pairing pipeline. The miRNA target filter in Qiagen’s IPA software was used for pairing of the miR-seq and RNA-seq data sets. The horizontal red line indicates where threshold cutoffs were used, and blue text denotes key steps in the analysis process. See the text for details.

The ingenuity pathway analysis (IPA) miR target filter was used to predict mRNA targets. IPA contains ~1.5 million miR targeting interactions and incorporates experimentally validated miR interactions from TarBase and miRecords, predicted mRNA targets from TargetScan, and miR-related findings manually curated from the published literature. Targeting information was available for 52 of the 78 differentially expressed miRs and yielded 13,217 potential mRNA targets. To restrict the potential targets to just those RNAs detected by RNA-seq, we incorporated the RNA-seq data set into the miR target filter analysis pipeline. To specifically identify mRNAs inversely correlated in expression with corresponding miRs, we then used an expression-pairing filter. Integration of the RNA-seq data, along with the inverse correlation expression-pairing filter, reduced the number of potential mRNA targets to 1,456 for 51 differentially expressed miRs. This corresponds to an average of 29 potential mRNA targets per individual miR. A schematic of the miR-mRNA expression-pairing pipeline is shown in Fig. 4. The top 10 most up- and downregulated miRs resulting from the miR-mRNA pairing analysis are shown in Table 1, and the full results of this analysis are shown in Table S4.

**TABLE 1:** Top 10 up- and downregulated miRs identified in the miR-mRNA pairing analysis with the potential for functional importance in HPV16 E6/E7-expressing HFKs

miR	FC (E6E7/C)^a^	*P* value	No. of target genes	Top 5 target gene products
Upregulated miRs/downregulated targets				
miR-363-3p	96.11	≥1.00E-15	92	KLK12, SLC6A14, STEAP4, GRHL1, ACAN
miR-9-5p	20.46	≥1.00E-15	103	WFDC12, SMPD3, CALB2, TNNT2, MUC15
miR-20b-5p	9.99	1.02E-08	103	KRT23, CRCT1, ATP12A, KLK7, SPACA4
miR-450a-5p	7.48	1.42E-06	8	PCDHGB7, ZNF365, IGLON5, ZNF385A, DUSP10
miR-542-3p	7.10	2.02E-04	39	ALDH3B2, CST6, MUC15, PPP2R2C, SPSB4
miR-155-5p	7.08	≥1.00E-15	50	MAFB, CSF2RB, INPP5D, SHANK2, GJA5
miR-33b-3p	6.07	1.42E-06	46	LCE3D, WFDC12, LCE3E, TMPRSS13, TGM5
miR-4435	5.59	6.62E-05	75	CNFN, SCNN1B, RNF222, KLK11, TMPRSS13
miR-195-5p	5.56	3.54E-03	123	CEACAM6, HMOX1, ZNF750, RASGEF1B, LYPD5
miR-30b-3p	5.34	3.37E-02	113	PI3, PLA2G4E, ALDH3B2, KLK7, HOPX
miR-335-5p	5.27	≥1.00E-15	40	KPRP, XKRX, INPP5D, CTSV, SLC15A1
miR-199b-5p	5.07	5.21E-03	53	CRCT1, KLK7, A2ML1, RPS10-NUDT3, TSPYL6
Downregulated miRs/upregulated targets				
miR-1249	−9.19	3.66E-07	24	ICAM5, FGFBP3, CRIP2, CERS1, CNTD2
miR-203	−8.93	≥1.00E-15	85	TNFRSF8, GLYATL2, GABRA5, NEK2, Ina
miR-34a-5p	−8.47	≥1.00E-15	111	TLX2, MCIDAS, NUP210, FOXR2, IL21R
miR-485-3p	−7.78	9.87E-06	64	LY75, PRRX1, SHISA2, RIPPLY3, HMMR
miR-34c-3p	−4.25	2.07E-06	37	LY75-CD302, SMIM10, PEG3, WDR76, TMEM56
miR-193b-3p	−3.80	≥1.00E-15	58	KLRG2, TAF7L, SPATA31D1, PNMA3, CAMK2N2
miR-197-3p	−3.62	1.46E-10	65	MEIOB, ZNF853, BTNL9, GABRA5, TFR2
miR-323a-3p	−3.41	2.40E-02	18	PLA2G3, HENMT1, PLPP2, ANKRD20A4, ZFPM2
miR-485-5p	−3.27	3.28E-03	120	CLDN11, PNMAL2, THY1, TMEM200B, GOLGA6L1
miR-328	−3.13	3.28E-03	118	GCK, SYNGR3, LRRC10B, ISM2, LCK

^a^ FC, fold change; C, control.

10.1128/mBio.02170-16.4 TABLE S4  Full results of miR-mRNA pairing analysis. Download Table S4, XLSX file, 0.2 MB. Copyright © 2017 Harden et al. 2017 Harden et al. This content is distributed under the terms of the Creative Commons Attribution 4.0 International license.

Of the 1,456 potential miR targets identified, 711 mRNAs (49%) are potentially targeted by more than one miR. Of these, 349 mRNAs are potentially targeted by two, 182 by three, 90 by four, 46 by five, and 22 by six miRs. All mRNAs potentially targeted by seven or more miRs are listed in Table S5. In particular, transcriptional repressor GATA binding 1 (TRPS1) is potentially targeted by 10 miRs and the ABL proto-oncogene 2 nonreceptor tyrosine kinase (ABL2) is potentially targeted by 12 miRs. The average fold change in the number of miRs potentially targeting ABL2 is 14.34 and the range of miR expression is between 3- and 96-fold. We also examined TRPS1, targeted by 10 miRs, and observed an average change in targeting miRs of 4.7-fold with a range of 3- to 10-fold. On the basis of these data, it does not appear that all of the miRs targeting a particular mRNA are increased or decreased to a similar degree.

10.1128/mBio.02170-16.5 TABLE S5  RNAs identified via miR-mRNA pairing analysis to be potentially targeted by two or more miRs. Download Table S5, DOCX file, 0.1 MB. Copyright © 2017 Harden et al. 2017 Harden et al. This content is distributed under the terms of the Creative Commons Attribution 4.0 International license.

Overall, this data-driven integration of the miR-seq and RNA-seq data sets revealed that the expression of 67.8% (1,456/2,149) of the potential target mRNAs is inversely correlated with the expression of the respective miRs, suggesting that these mRNAs may represent biologically relevant targets of the corresponding miRs.

### Identification of miRs with the potential to regulate targets in HFKs expressing HPV16 E6/E7.

On the basis of integrative analysis of the miR-seq and RNA-seq data described above, we next generated a list of miRs that may have functional importance in HPV16 E6/E7-expressing HFKs (Table 1; see Table S4). This list was curated on the basis of our initial miR-seq data and incorporates the miR-mRNA pairing analysis described above. Some highly differentially expressed miRs had a large number of potential targets identified via the miR-mRNA pairing analysis. An example is miR-9-5p, which is upregulated in HFKs expressing HPV16 E6/E7 by 20-fold and has 102 potential targets. Likewise, some miRs are less dramatically differentially expressed in HFKs expressing HPV16 E6/E7 and have few potential targets. For example, miR-577 is upregulated 3.5-fold in HPV16 E6/E7-expressing HFKs and has eight potential targets. However, in other cases, the extent of differential miR expression did not correlate with the number of potential mRNA targets modulated by a given miR. Some highly differentially expressed miRs were paired with very few potential mRNA targets in HPV16 E6/E7-expressing HFKs. In particular, miR-450a-5p was upregulated 7-fold in HFKs expressing HPV16 E6/E7 but had only eight potential targets. In contrast, other less dramatically differentially expressed miRs were paired with a large number of potential targets. For example, miR-4532 was upregulated only 3-fold in HPV16 E6/E7-expressing HFKs but could be paired with 90 potential mRNA targets. Hence, our integration of the miR-seq and RNA-seq data sets allowed the identification of miRs with the greatest potential for miR-mediated mRNA target regulation, rather than just a set of differentially expressed miRs.

**FIG 5:**
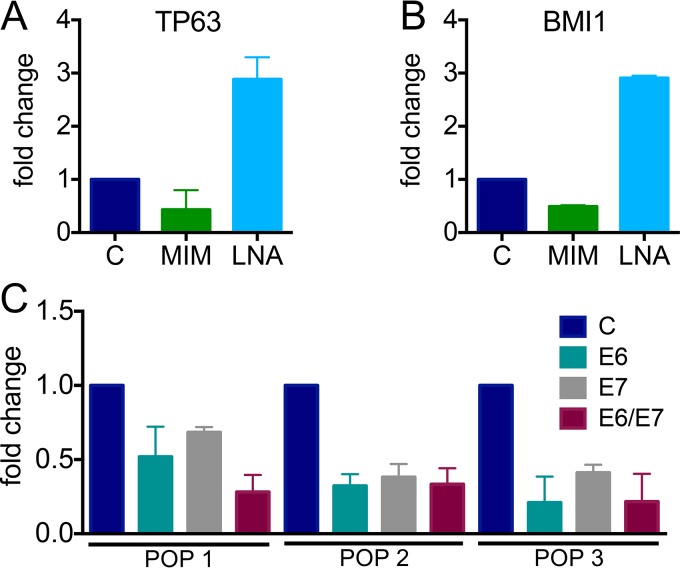
Modulation of miR-203a-3p targets and analysis of miR-203a-3p expression. Effects of a miR-203a-3p mimic (MIM, green) or an LNA inhibitor (light blue) on TP63 (A) and BMI1 (B) levels in HPV16 E6/E7-expressing HFKs. Expression of 18S rRNA was used as an internal control, and values were normalized to a negative control (C) mimic or LNA. TP63 and BMI1 expression was assessed by RT-PCR. (C) Expression of miR-203a-3p in three independently derived HFK populations expressing HPV16 E6, E7, or E6/E7 or a control vector via TaqMan miR assay. Expression of the noncoding snRNA U6 spliceosomal RNA was used as an internal control. Results represent averages of at least three independent experiments.

One example of a well-studied miR that we validated from our curated list is miR-203a-3p, which is thought to act as a “switch” between epithelial proliferation and differentiation by targeting TP53-related TP63 (37). The Laimins laboratory first showed that HPVs block the induction of miR-203a-3p during differentiation through E7-mediated interference of the mitogen-activated protein kinase (MAPK)/protein kinase C pathway and that miR-203a-3p inhibition was necessary for HPV genome amplification upon differentiation, as well as for long-term maintenance of HPV episomes (38). The McCance laboratory also investigated miR-203a-3p, reporting that miR-203a-3p levels are reduced by E6 via abrogation of TP53 (39). Our miR-seq and reverse transcription-quantitative PCR (RT-qPCR) data suggest that miR-203a-3p levels are decreased by both HPV16 E6 and E7 (Fig. 5). Integration of the miR-seq and RNA-seq data revealed 85 potential targets of miR-203a-3p in HPV16 E6/E7-expressing HFKs. We examined two canonical miR-203a-3p targets, TP63 and BMI1 (40). Using a miR mimic to overexpress miR-203a-3p, we restored miR-203a-3p levels in HPV16 E6/E7-expressing HFKs and observed decreased TP63 and BMI1 steady-state mRNA levels. When we inhibited miR-203a-3p in HFKs expressing HPV16 E6/E7 via a locked nucleic acid (LNA) inhibitor, we were able to further decrease miR-203a-3p levels, which resulted in higher TP63 and BMI1 mRNA levels (Fig. 5). Taken together, our data show that both HPV16 E6 and E7 function to reduce miR-203a-3p levels.

### Potential miR targets are involved in unique pathways compared to overall gene expression changes in HPV16 E6/E7-expressing HFKs.

To categorize pathways relevant to the observed changes in miR expression as a result of HPV16 E6/E7 expression, we used the core analysis function of IPA. This analysis identifies relationships, mechanisms, functions, and pathways of relevance to a particular data set. We compared core analyses of the miR-modulated mRNAs identified in the miR-mRNA pairing analysis with the mRNA expression changes identified by RNA-seq. Both data sets were found to be associated with “cancer” and “reproductive system disease,” as well as “cellular movement” and “cell morphology.” However, some predicted molecular and cellular functions, including “cellular development,” “morphology,” and “growth and proliferation” were specifically associated with changes in miR-targeted mRNAs (Table S6). Additionally, the highest scoring predicted upstream regulators were different between the two data sets (Table S7). Overall, these data show that HPV16 E6/E7-regulated mRNAs that are candidate targets of miR modulation are associated with some biological activities and biochemical pathways that are distinct from expression changes that are not directly modulated by miR expression.

10.1128/mBio.02170-16.6 TABLE S6  Molecular and cellular functions identified via IPA core analysis associated with the RNA-seq and potential miR target RNA data sets. Download Table S6, DOCX file, 0.1 MB. Copyright © 2017 Harden et al. 2017 Harden et al. This content is distributed under the terms of the Creative Commons Attribution 4.0 International license.

10.1128/mBio.02170-16.7 TABLE S7  Predicted upstream regulators identified via IPA core analysis based on gene expression changes observed in the RNA-seq and potential miR target RNA data sets. Download Table S7, DOCX file, 0.1 MB. Copyright © 2017 Harden et al. 2017 Harden et al. This content is distributed under the terms of the Creative Commons Attribution 4.0 International license.

## DISCUSSION

The high-risk HPV E6 and E7 proteins reprogram the infected host cell to allow for viral genome replication in growth arrested, terminally differentiated epithelial cells and are the main drivers of cell transformation that ultimately lead to HPV-associated cancers. Since miRs modulate levels and/or translation of multiple host mRNAs that regulate a variety of biological activities, they are particularly attractive targets for the HPV E6 and E7 proteins. In this study, we used deep sequencing to examine miR expression and also examined changes in RNA expression as a result of HPV16 E6/E7 in parallel. Integrating the two data sets, we identified miRs modulated by expression of HPV16 E6/E7 that may have functional implications in high-risk HPV biology.

We observed 67.8% of potential target RNAs inversely correlated with expression of their respective miRs, suggesting the potential for miR-mediated regulation of these RNAs. This value agrees closely with estimations that 60% of all mRNAs are controlled by miRs (11), consistent with the notion that miR regulation is the most abundant mode of posttranscriptional regulation of gene expression (41).

Bioinformatic analyses identified several cellular processes that were significantly targeted by miR-modulated mRNAs. Additional analyses with IPA revealed canonical pathways, including cyclins, cell cycle regulation (*z* score of 2.33), and estrogen-mediated S-phase entry (*z* score of 2.24), to be significantly activated and aryl hydrocarbon reception signaling to be significantly inhibited (*z* score of −2.45) in the RNA-seq data set of all RNAs altered by HPV16 E6/E7 expression. In contrast, ATM signaling (*z* score of 2.12) was significantly activated in HPV16 E6/E7-expressing HFKs on the basis of analysis of miR-modulated RNAs. These results suggest that miR-modulated RNAs in HPV16 E6/E7 HFKs are involved in distinct canonical pathways that are relevant in the context of HPV biology and imply that HPV16 E6/E7 regulation of cellular miRs contributes to the biological activities of these two proteins.

A total of 49% of the potential target RNAs are potentially modulated by multiple miRs. Analysis of RNAs targeted by more than one miR indicates that endothelian-1 signaling (*z* score of −2.11), p38 MAPK signaling (*z* score of −2.12), and G_1_/S checkpoint regulation (*z* score of −2.24) are significantly inhibited and that the ATM signaling pathway is significantly activated (*z* score of 2.24). RNAs potentially targeted by just one miR in our study were upregulated 3.9-fold and downregulated 13.0-fold, on average, whereas RNAs potentially targeted by more than one miR were upregulated 3.4-fold and downregulated 5.7-fold, on average. This suggests that many miR-modulated RNAs may also be regulated by other mechanisms, consistent with the notion that miRs act to “fine-tune” gene expression.

Our analysis showed that more miRNAs are upregulated than downregulated in response to HPV16 E6/E7 expression. While reduced levels of miRNAs are often observed in tumors because of genetic loss, epigenetic silencing, defects in miRNA biogenesis, or widespread transcriptional repression (42, 43), our results may be explained by the fact that our experimental system more closely mimics an HPV-associated premalignant lesion than a late-stage invasive carcinoma. Nevertheless, many of the miR expression changes detected in HPV16 E6/E7-expressing HFKs were also observed in HPV^+^ head and neck squamous cell carcinoma cell lines (44) and tumors (45). We also observed changes in miR expression that have been detected in HPV^+^ anal carcinomas (46), vulvar cancers (47), and penile squamous cell carcinoma (48). A comparison of our data to miR expression in HPV-associated human biopsy specimens are detailed in Tables S8 and S9. These results suggest that miR alterations in HPV-associated tumors are likely caused by HPV E6/E7 expression and that these miR-mRNA pairs may be potential “drivers” of HPV carcinogenesis. Our results also indicate that a core set of miRNAs may be altered in all HPV-associated epithelial cancers as a result of HPV16 E6/E7 expression, whereas some miRNAs may be specific to an HPV-associated cancer of a particular anatomical site.

10.1128/mBio.02170-16.8 TABLE S8  Comparison of trends in miR expression in HFKs expressing HPV16 E6/E7 with previously described HPV-associated miRs. Download Table S8, DOCX file, 0.2 MB. Copyright © 2017 Harden et al. 2017 Harden et al. This content is distributed under the terms of the Creative Commons Attribution 4.0 International license.

10.1128/mBio.02170-16.9 TABLE S9  Comparison of trends in miR expression in HFKs expressing HPV16 E6/E7 with a study by Wang et al. (1,7). Download Table S9, DOCX file, 0.1 MB. Copyright © 2017 Harden et al. 2017 Harden et al. This content is distributed under the terms of the Creative Commons Attribution 4.0 International license.

Modulation of cellular miR levels by HPV gene expression has been previously investigated by other groups (17, 18). We examined miR expression in more uniform populations of undifferentiated HFKs, allowing us to identify miRs that are likely modulated directly as a consequence of HPV16 E6/E7 expression rather than representing the expansion of proliferating, undifferentiated cells in E6/E7-expressing raft cultures. Simultaneous miR-seq and RNA-seq enabled us to investigate in detail the potential influence of miR regulation on overall gene expression in HPV16 E6/E7-expressing HFKs. Tables S8, S9, and S10 compare our data with those of other studies of HPV-associated miRs in the literature. We hypothesize that many of the differences in miR expression that we observed are the result of analyzing undifferentiated human epithelial cells, whereas most other studies analyzed differentiating cells. Additional differences may be due to differences in HPV type or analysis of the effect of whole HPV genomes compared to our study, which focused only on effects of HPV16 E6 and/or E7 on miRs.

10.1128/mBio.02170-16.10 TABLE S10  Comparison of trends in miR expression in HFKs expressing HPV16 E6/E7 with a study by Gunasekharan et al. (1,8). Download Table S10, DOCX file, 0.1 MB. Copyright © 2017 Harden et al. 2017 Harden et al. This content is distributed under the terms of the Creative Commons Attribution 4.0 International license.

While the focus of our study was on alterations in miRs resulting from the expression of both HPV16 E6 and E7, we also performed miR-seq of HFKs expressing HPV16 E6 or E7 alone to understand the consequences of individual oncoproteins on miR expression. Given that the TP53 and the E2F pathways are key targets of HPV16 E6 and E7, respectively, we considered the possibility that some of the miRs regulated by HPV16 E6 or E7 may be TP53- or E2F-responsive miRs. The miR-106b~25 cluster is known to be regulated by E2F family members (49), and a member of that cluster, miR-25-5p, is one of the top miRs upregulated by HPV16 E7. Additionally, the miR-15b~16-2 cluster is an E2F target (50) and all three members of that cluster, miR-15b-5p, -16-5p, and -16-2-3p, are on our list of HPV16 E7-upregulated miRs. The TP53 tumor suppressor can transcriptionally activate miR genes, as is the case for the miR-34 family and others (51–54). Both miR-34a-5p and -34c-3p are downregulated in HPV16 E6-expressing HFKs. Additionally, TP53 can activate the processing of specific miRs, such as miR-143-3p (55, 56), which was on our list of HPV16 E6-downregulated miRs. Other HPV16 E6- and/or E7-modulated miRs have not been identified as TP53 or E2F responsive, suggesting that HPV16 E6 and E7 may also alter miR expression through other mechanisms.

We also compared our list of top upregulated and downregulated miRs in HPV16 E6- or E7-expressing HFKs to a miR analysis performed by the Khan laboratory (9). Several miRs, for example, miR-100-3p, were also found to be upregulated by HPV16 E7, whereas other miRs showed different trends of expression. We also compared our results to those of a study examining miR expression resulting from the expression of HPV18 E6 or E7 (17). Our data agree with their observation of downregulation of miR-34a-5p by E6 and upregulation of miR-25-5p by E7, as well as the finding that modulation of the expression of some miRs can be attributed to one or both oncoproteins.

Our study showed that expression of HPV16 E6/E7 in HFKs not only changes the expression of individual miRs but also alters the expression of entire groups of genomically clustered miRs. Of interest, we observed some of the same miR clusters altered by HPV16 E6/E7 expression as in studies of cervical cancer (57). HPV16 E6/E7 modulates both tumor-suppressive and oncogenic miR clusters. For example, HPV16 E6/E7 upregulates all of the miRs of the oncogenic miR-106b~25 cluster (58, 59) and downregulates all of the miRs of the tumor-suppressive miR-34b~34c cluster (reviewed in reference 60).

Most of the early studies on miRs in cancer have focused on a single miR and the modulation of a single target mRNA. While these studies were useful, this paradigm of research in the miR field has now been mostly replaced with studies that analyze the global landscape of miR expression and use integrative methods to investigate the potential effects of these alterations on cellular processes. Additionally, human cells encode ≥2,500 mature miRs and a single miR can regulate the expression and/or translation of hundreds of RNA targets. Therefore, aberrant miR expression will influence a multitude of target transcripts, causing alterations in multiple signaling pathways. Moreover, many mRNAs are subject to regulation by multiple miRs.

Our study shows that high-risk HPV E6/E7 expression in normal human cells causes a dramatic rewiring of cellular gene expression and that modulation of cellular miR expression plays an important role in this process. A large percentage of the RNAs expressed in HPV16 E6/E7-expressing keratinocytes are potentially targeted by miRs that are modulated by E6/E7 expression. Genes involved in specific cellular processes and pathways, including cell cycle regulation and ATM signaling, seem to be selectively regulated by miRs. Moreover, our study has also identified some miRs that have been previously reported to be dysregulated in HPV-associated lesions and cancers as targets of the HPV E6 and E7 oncoproteins.

### Accession number.

The miRseq and RNA seq data sets generated for this study have been deposited at GEO (Gene Expression Omnibus) under accession number GSE92496.

## MATERIALS AND METHODS

### Cell culture.

HFKs were isolated from a pool of deidentified newborn foreskin samples and transduced with LXSN-based recombinant retroviruses encoding HPV16 E6, E7, or both oncogenes or a control LXSN vector as previously described (61). The two independent HFK populations used in this study were generated from two distinct pools of human foreskin samples. Donor-, passage-, and density-matched HFK populations were used in all experiments.

### RNA isolation.

Large (≥200-nt) and small (<200-nt) RNAs were prepared for sequencing with the mirVana miRNA isolation kit (Ambion, Life Technologies, Inc.) according to the manufacturer’s protocol. For RT-qPCR experiments, total RNA was isolated with the miRNeasy minikit (Qiagen) as described in the manufacturer’s instructions.

### miR-seq.

Small RNA libraries were prepared from small RNA with the TruSeq small RNA Library Preparation kit (Illumina) as described in the manufacturer’s sample preparation guide. Gel-purified small RNA cDNA libraries were quantified with the Qubit 2.0 Fluorometer (Invitrogen, Life Technologies, Inc.), diluted to a final concentration of 10 nM, and pooled in equimolar amounts prior to cluster generation. Single-read (SR) sequencing (1 to 2 million 50-bp PE reads) was performed with the Illumina MiSeq Sequencing System (Illumina).

### miR-seq data analysis.

Postprocessing of the miR-seq reads from each sample was performed according to the HudsonAlpha Genomic Services Laboratory (GSL) unique in-house pipeline as previously described (62). The differential expression of miRs was calculated on the basis of the difference (cutoff, ±3.0-fold or more) observed between different groups (control HFKs versus HFKs plus HPV16 E6/E7). The *P* value of differentially expressed miRs was estimated via *z* score by using a Benjamini-Hochberg FDR corrections of 0.05 (63).

### RNA-seq.

The concentration and integrity of the isolated large (≥200-nt) RNA were estimated with a Qubit 2.0 Fluorometer (Invitrogen, Life Technologies, Inc.) and an Agilent 2100 Bioanalyzer (Applied Biosystems, Life Technologies, Inc.), respectively. Five hundred nanograms of large RNA was used for downstream RNA-seq processing. First, rRNA was removed with the Ribo-Zero Magnetic Gold (Yeast) kit (Epicentre, Illumina) according to the manufacturer’s recommended protocol. The RNA was then fragmented and primed for first-strand synthesis with the NEBNext RNA First Strand Synthesis Module (New England Biolabs). Second-strand synthesis was performed with the NEBNext RNA Second Strand Synthesis Module.

Samples were prepared with the NEBNext DNA Library Prep Master Mix Set for Illumina, with slight modifications. Briefly, end repair was performed, followed by A tailing and custom adapter ligation. Samples were then individually bar coded with GSL primers and amplified by 12 cycles of PCR. Library quantity was assessed with a Qubit 2.0 fluorometer, and library quality was estimated with a DNA 1000 chip on an Agilent 2100 Bioanalyzer. Further quantification of the final libraries for downstream sequencing applications was done with the qPCR-based KAPA Biosystems Library Quantification kit (Kapa Biosystems). Each library was diluted to a final concentration of 12.5 nM and pooled in equimolar amounts prior to clustering. PE sequencing (50 million 100-bp PE reads) was performed with the Illumina HiSeq 2500 Sequencing System (Illumina).

### Processing and analysis of RNA-seq reads.

Downstream analysis of the sequenced reads from each sample was performed with a unique in-house pipeline designed by GSL. Briefly, quality control checks of raw sequence data from each sample were performed with FastQC (Babraham Bioinformatics). Raw reads were then mapped to the reference human genome hg19 with TopHat v2.0 (64, 65) with two mismatches allowed and other default parameters. The alignment metrics of the mapped reads were estimated with SAMtools (66). Aligned reads were then imported into the commercial data analysis platform, Avadis NGS (Strand Scientifics). After quality inspection, the aligned reads were filtered on the basis of read quality metrics where reads with base quality scores of <30, alignment scores of <95, and mapping qualities of <40 were removed. The remaining reads were then filtered on the basis of read statistics, where missing mates and translocated, unaligned, and flipped reads were removed. The list of reads was then filtered to remove duplicates. Samples were grouped, and quantification of transcript abundance in this final read list was performed with trimmed means of M values (67) as the normalization method. Differential expression of RNAs was calculated on the basis of the difference (cutoff, ±2.0-fold or greater) observed between defined conditions. The *P* value of the differentially expressed RNAs was estimated by *z* score calculations with a Benjamini-Hochberg FDR correction of 0.05 (63). IPA software (Qiagen) was used to analyze the unique canonical pathways, biological functions, and networks affected.

### Integration of RNA-seq and miR-seq data.

Differentially expressed miRs identified via miR-seq that met threshold cutoffs (change, ±3.0-fold or greater; FDR, ≤0.05) were uploaded into IPA (Qiagen) and analyzed with the miR target filter. This filter displays experimentally validated and predicted mRNA targets from TargetScan, TarBase, miRecords, and the Ingenuity Knowledge Base for each miR in the data set. Differentially expressed RNAs identified by RNA-seq that met threshold cutoffs (change, ±2.0-fold or greater; FDR, ≤0.05) were then uploaded with the “add/replace mRNA data set” function. Using the “expression-pairing” feature, only potential targets differentially expressed in the RNA-seq data are shown; all other potential targets are filtered out. To further refine the data, the “inverse correlation” filter was used to focus on changes in potential targets that are inversely correlated with changes in the corresponding miR.

### RT-qPCR.

For miR RT-qPCR, total RNA was reverse transcribed with the TaqMan miR RT kit (Applied Biosystems, Life Technologies, Inc.) as described in the manufacturer’s protocol, with miR-specific, stem-loop primers (Applied Biosystems, Life Technologies, Inc.). TaqMan miR assays (Applied Biosystems, Life Technologies, Inc.) were used to detect mature miRs by the comparative *C*_*T*_ method with the StepOnePlus real-time PCR system (Thermo Fisher Scientific). Assay IDs 000390, 000391, 002367, and 000507 were used to detect miR-15b-5p, -16-5p, -193b-3p, and -203a-3p, respectively. RT-qPCR assays were performed in triplicate, and the noncoding small nuclear RNA (snRNA) U6 (assay ID, 001973) was used as an endogenous small-RNA control.

For RT-qPCR of miR targets, following RNA isolation, total RNA was DNase treated with the TURBO DNA-free kit. DNase-treated total RNA was then reverse transcribed with TaqMan RT reagents (Life Technologies, Inc., Applied Biosystems). TaqMan assays for TP63 (assay ID, Hs00978343_m1) and BMI1 (assay ID, Hs00995536_m1) were used to detect targets by the comparative *C*_*T*_ method with the StepOnePlus real-time PCR system (Thermo Fisher Scientific). RT-qPCR assays were performed in triplicate, and 18S rRNA was used as an internal control.

### miR mimics and inhibitors.

Overexpression of miR-203a-3p was achieved with a miRCURY LNA miR mimic (472239-001; Exiqon). A negative-control miR mimic (miRCURY LNA miR mimic negative control 479903-001; Exiqon) with the same design features as the miRCURY LNA miR mimics and no homology to any known miR or mRNA sequences in mice, rats, or humans was used as a negative control for overexpression. Inhibition of miR-203a-3p was accomplished with a miRCURY LNA miR power inhibitor (4100339-101; Exiqon). A miRCURY LNA miR inhibitor control (199006-101; Exiqon) that is similar in sequence length and LNA design with no homology to any known miR or mRNA sequence in the mouse, rat, or human genome was used as a negative control for miR inhibition.

### Transfection of miR mimics and inhibitors.

HFKs were transfected with miR mimics and inhibitors by using Lipofectamine 2000 (Invitrogen, Life Technologies, Inc.) as described in the manufacturer’s instructions, with some modifications. To achieve optimal overexpression of miRs with a miRCURY LNA miR mimic (Exiqon), 0.05 nM mimic was transfected and samples were harvested at 24 h posttransfection. To achieve optimal knockdown of miRs with a miRCURY LNA miR power inhibitor (Exiqon), 20 nM inhibitor was transfected and samples were harvested at 48 h posttransfection. The same amount of control mimic or inhibitor was transfected, and control samples were harvested at 24 or 48 h posttransfection, respectively. As an additional control, HFKs were treated with the transfection reagents alone. RT-qPCR for known miR targets was used to confirm successful miR overexpression or knockdown.
