# Supplementary material for: Modulation of microRNA-mRNA Target Pairs by Human Papillomavirus 16 Oncoproteins
Source: mBio. 2017 Jan 3;8(1):e02170-16. doi: 10.1128/mBio.02170-16 (PMC5210503; doi:10.1128/mBio.02170-16)
Supplement: TABLE S1 [file mbo006163134st1.docx]

**Table S1. miRs modulated in HFKs with expression of individual viral oncoproteins (**threshold cut-offs: ≥ 10 reads; FDR ≤ 0.05)

| 16E7: Up-Regulated miRs | |  | 16E6: Up-Regulated miRs | |
| --- | --- | --- | --- | --- |
| miR | FC |  | miR | FC |
| miR-542-3p^a^ | 7.86 |  | miR-363-3p^b^ | 44.68 |
| miR-100-3p | 5.55 |  | miR-9-5p^b^ | 15.16 |
| miR-345-5p | 5.37 |  | miR-335-3p^a^ | 13.57 |
| miR-7974^a^ | 5.11 |  | miR-335-5p | 10.13 |
| miR-181b-3p | 5.10 |  | miR-542-3p^a^ | 6.84 |
| miR-30b-3p | 4.74 |  | miR-450a-5p^b^ | 6.46 |
| miR-224-3p^a^ | 4.43 |  | miR-1271-5p | 6.06 |
| miR-25-5p | 4.22 |  | miR-450b-5p | 5.51 |
| miR-129-5p | 4.06 |  | miR-193a-5p | 4.91 |
| miR-16-2-3p^b^ | 4.06 |  | miR-7974^a^ | 4.78 |
| miR-15b-5p^a^ | 3.96 |  | miR-548w | 4.48 |
| miR-335-3p^a^ | 3.95 |  | miR-224-3p^a^ | 4.42 |
| miR-16-5p | 3.60 |  | miR-362-5p | 4.26 |
| miR-33b-3p^a^ | 3.52 |  | miR-33b-3p^a^ | 4.26 |
| miR-873-3p | 3.23 |  | miR-15b-5p^a^ | 4.10 |
|  |  |  |  |  |
| 16E7: Down-Regulated miRs | |  | 16E6: Down-Regulated miRs | |
| miR | FC |  | miR | FC |
| miR-197-3p^b^ | -2.01 |  | miR-34a-5p^b^ | -3.58 |
| miR-10a-3p | -1.97 |  | miR-487b-3p | -2.62 |
| miR-485-3p | -1.96 |  | miR-485-3p | -2.18 |
| miR-203a-3p | -1.75 |  | miR-328-3p | -2.08 |
| miR-328-3p | -1.73 |  | miR-642a-5p | -2.03 |
| miR-193b-3p | -1.63 |  | miR-34c-3p^b^ | -1.83 |
| miR-1249^b^ | -1.54 |  | miR-758-3p | -1.75 |
| miR-3607-3p | -1.54 |  | miR-410-3p | -1.72 |
| miR-3065-5p | -1.50 |  | miR-143-3p | -1.71 |
| miR-136-3p | -1.49 |  | miR-31-3p | -1.69 |
| miR-873-5p | -1.43 |  | let-7d-3p | -1.51 |
| miR-3065-3p | -1.30 |  | miR-193b-3p | -1.46 |
| let-7e-3p | -1.30 |  | miR-3065-3p | -1.42 |
| miR-132-3p | -1.25 |  | miR-136-3p | -1.41 |
| miR-221-3p | -1.10 |  | miR-3614-5p | -1.30 |

^a^miRs that are up or down-regulated by both HPV16 E6 and E7 individually and in HPV16 E6/E7 expressing HFKs

^b^miRs that are up or down-regulated by one oncoprotein and also up or down-regulated in HPV16 E6/E7 expressing HFKs
