# Supplementary material for: Modulation of microRNA-mRNA Target Pairs by Human Papillomavirus 16 Oncoproteins
Source: mBio. 2017 Jan 3;8(1):e02170-16. doi: 10.1128/mBio.02170-16 (PMC5210503; doi:10.1128/mBio.02170-16)
Supplement: TABLE S2 [file mbo006163134st2.docx]

**Table S2. miR clusters modulated by expression of HPV16 E6/E7**

| **Clustered miRs^a^** | **Chromosome Location** | **FC (E6E7/C)** |
| --- | --- | --- |
| miR-374c-5p | chrX: 74218549-74218618 [+] | -1.0 |
| **miR-374b-3p** | chrX: 74218547-74218618 [-] | 3.5 |
| miR-374b-5p | chrX: 74218547-74218618 [-] | 1.6 |
| miR-421 | chrX: 74218377-74218461 [-] | 2.1 |
|  | | |
| miR-532-5p | chrX: 50003148-50003238 [+] | -1.2 |
| miR-532-3p | chrX: 50003148-50003238 [+] | -1.0 |
| miR-188-5p | chrX: 50003503-50003588 [+] | -1.4 |
| miR-500a-5p | chrX: 50008431-50008514 [+] | 1.7 |
| **miR-362-5p** | chrX: 50008964-50009028 [+] | 3.7 |
| miR-362-3p | chrX: 50008964-50009028 [+] | -1.0 |
| miR-501-5p | chrX: 50009722-50009805 [+] | 1.2 |
| miR-500b | chrX: 50010672-50010750 [+] | -1.0 |
| miR-660-5p | chrX: 50013241-50013337 [+] | -1.4 |
| miR-660-3p | chrX: 50013241-50013337 [+] | -1.6 |
| miR-502-5p | chrX: 50014598-50014683 [+] | -1.0 |
| miR-502-3p | chrX: 50014598-50014683 [+] | -1.2 |
|  | | |
| **miR-106a-5p** | chrX: 134170198-134170278 [-] | 5.9 |
| miR-18b-5p | chrX: 134170041-134170111 [-] | 2.3 |
| **miR-20b-5p** | chrX: 134169809-134169877 [-] | 10.0 |
| miR-20b-3p | chrX: 134169809-134169877 [-] | 1.3 |
| miR-19b-2-5p | chrX: 134169671-134169766 [-] | -1.0 |
| miR-92a-2-5p | chrX: 134169538-134169612 [-] | 1.3 |
| **miR-363-3p** | chrX: 134169378-134169452 [-] | 96.1 |
|  | | |
| miR-424-5p | chrX: 134546614-134546711 [-] | 1.0 |
| miR-503 | chrX: 134546328-134546398 [-] | 1.3 |
| miR-542-5p | chrX: 134541341-134541437 [-] | 2.1 |
| **miR-542-3p** | chrX: 134541341-134541437 [-] | 7.1 |
| **miR-450a-2-5p** | chrX: 134540508-134540607 [-] | 7.5 |
| miR-450a-1-5p | chrX: 134540341-134540431 [-] | 7.5 |
| miR-450b-5p | chrX: 134540185-134540262 [-] | 2.7 |
|  | | |
| hsa-let-7a-5p | chr9: 94175957-94176036 [+] | 1.0 |
| hsa-let-7a-3p | chr9: 94175957-94176036 [+] | 1.6 |
| hsa-let-7f-1 | chr9: 94176347-94176433 [+] | -1.0 |
| hsa-let-7d-5p | chr9: 94178834-94178920 [+] | 1.2 |
| **hsa-let-7d-3p** | chr9: 94178834-94178920 [+] | -3.5 |
|  | | |
| miR-3154 | chr9: 128244947-128245030 [-] | -1.0 |
| **miR-199b-5p** | chr9: 128244721-128244830 [-] | 5.1 |
| miR-199b-3p | chr9: 128244721-128244830 [-] | 1.6 |
|  | | |
| miR-30d-5p | chr8: 134804876-134804945 [-] | 1.2 |
| miR-30d-3p | chr8: 134804876-134804945 [-] | 1.6 |
| **miR-30b-3p** | chr8: 134800520-134800607 [-] | 5.3 |
|  | | |
| **miR-550a-5p** | chr7: 30289794-30289890 [+] | 4.8 |
| miR-550a-3p | chr7: 30289794-30289890 [+] | 2.0 |
| miR-550b-1 | chr7: 30289794-30289890 [-] | -1.0 |
|  | | |
| miR-106b-5p | chr7: 100093993-100094074 [-] | 2.4 |
| miR-106b-3p | chr7: 100093993-100094074 [-] | 1.4 |
| miR-93-5p | chr7: 100093768-100093847 [-] | 1.9 |
| miR-93-3p | chr7: 100093768-100093847 [-] | 1.6 |
| **miR-25-5p** | chr7: 100093560-100093643 [-] | 5.1 |
| miR-25-3p | chr7: 100093560-100093643 [-] | 2.7 |
|  | | |
| **miR-29b-1-5p** | chr7: 130877459-130877539 [-] | 5.4 |
| miR-29b-1-3p | chr7: 130877459-130877539 [-] | 1.6 |
| miR-29a-5p | chr7: 130876747-130876810 [-] | 1.6 |
| miR-29a-3p | chr7: 130876747-130876810 [-] | 1.6 |
|  | | |
| **miR-15b-5p** | chr3: 160404588-160404685 [+] | 4.2 |
| **miR-15b-3p** | chr3: 160404588-160404685 [+] | 6.4 |
| **miR-16-2-3p** | chr3: 160404745-160404825 [+] | 6.0 |
|  | | |
| miR-191-5p | chr3: 49020618-49020709 [-] | 1.8 |
| **miR-191-3p** | chr3: 49020618-49020709 [-] | 4.8 |
| miR-425-5p | chr3: 49020148-49020234 [-] | 1.5 |
| miR-425-3p | chr3: 49020148-49020234 [-] | 1.4 |
|  | | |
| **miR-642a-5p** | chr19: 45674928-45675024 [+] | -3.1 |
| miR-642b-5p | chr19: 45674932-45675008 [-] | -1.0 |
| miR-642b-3p | chr19: 45674932-45675008 [-] | -1.0 |
|  | | |
| **miR-212-5p** | chr17: 2050271-2050380 [-] | 8.2 |
| **miR-212-3p** | chr17: 2050271-2050380 [-] | 2.9 |
| **miR-132-5p** | chr17: 2049908-2050008 [-] | 3.0 |
|  | | |
| **miR-33b-5p** | chr17: 17813836-17813931 [-] | 3.2 |
| **miR-33b-3p** | chr17: 17813836-17813931 [-] | 6.1 |
| miR-6777 | chr17: 17813480-17813545 [-] | not detected |
|  | | |
| miR-497-5p | chr17: 7017911-7018022 [-] | -3.5 |
| **miR-195-5p** | chr17: 7017615-7017701 [-] | 5.6 |
| miR-195-3p | chr17: 7017615-7017701 [-] | 1.6 |
|  | | |
| **miR-193b-5p** | chr16: 14303967-14304049 [+] | -1.4 |
| **miR-193b-3p** | chr16: 14303967-14304049 [+] | -3.8 |
| miR-365a-5p | chr16: 14309285-14309371 [+] | -1.8 |
| miR-365a-3p | chr16: 14309285-14309371 [+] | 1.2 |
|  | | |
| miR-5587-5p | chr16: 535316-535368 [+] | -1.0 |
| miR-5587-3p | chr16: 535316-535368 [+] | -1.0 |
| **miR-3176** | chr16: 543277-543366 [+] | 3.1 |
|  | | |
| miR-381 | chr14: 101045920-101045994 [+] | -1.1 |
| miR-487b | chr14: 101046455-101046538 [+] | -2.3 |
| miR-539-5p | chr14: 101047321-101047398 [+] | -1.6 |
| miR-889 | chr14: 101047901-101047979 [+] | 1.1 |
| miR-544a | chr14: 101048658-101048748 [+] | -1.0 |
| miR-655 | chr14: 101049550-101049646 [+] | -1.0 |
| miR-487a | chr14: 101052446-101052525 [+] | 1.2 |
| miR-382-5p | chr14: 101054306-101054381 [+] | -1.0 |
| miR-382-3p | chr14: 101054306-101054381 [+] | -1.0 |
| miR-134 | chr14: 101054687-101054759 [+] | -1.4 |
| miR-668 | chr14: 101055258-101055323 [+] | -1.6 |
| **miR-485-5p** | chr14: 101055419-101055491 [+] | -3.3 |
| **miR-485-3p** | chr14: 101055419-101055491 [+] | -7.8 |
| miR-323b-5p | chr14: 101056219-101056300 [+] | -1.0 |
| miR-323b-3p | chr14: 101056219-101056300 [+] | -1.0 |
| miR-154-5p | chr14: 101059755-101059838 [+] | -1.1 |
| miR-154-3p | chr14: 101059755-101059838 [+] | -1.0 |
| miR-496 | chr14: 101060573-101060674 [+] | -1.0 |
| miR-377-3p | chr14: 101062050-101062118 [+] | -1.1 |
| miR-541-5p | chr14: 101064495-101064578 [+] | -1.0 |
| miR-541-3p | chr14: 101064495-101064578 [+] | -1.0 |
| miR-409-5p | chr14: 101065300-101065378 [+] | -1.5 |
|  | | |
| **miR-203a** | chr14: 104117405-104117514 [+] | -8.9 |
| miR-203b-5p | chr14: 104117418-104117503 [-] | -1.0 |
| miR-203b-3p | chr14: 104117418-104117503 [-] | 2.4 |
|  | | |
| miR-379-5p | chr14: 101022066-101022132 [+] | -1.0 |
| miR-411-5p | chr14: 101023325-101023420 [+] | 1.1 |
| miR-299-5p | chr14: 101023794-101023856 [+] | -1.0 |
| miR-380-5p | chr14: 101025017-101025077 [+] | -1.0 |
| miR-380-3p | chr14: 101025017-101025077 [+] | -1.0 |
| miR-1197 | chr14: 101025564-101025651 [+] | -1.0 |
| miR-323a-5p | chr14: 101025732-101025817 [+] | -1.0 |
| **miR-323a-3p** | chr14: 101025732-101025817 [+] | -3.4 |
| miR-758 | chr14: 101026020-101026107 [+] | -1.1 |
| miR-329-1 | chr14: 101026785-101026864 [+] | 1.3 |
| miR-329-2 | chr14: 101027100-101027183 [+] | 1.3 |
| miR-494 | chr14: 101029634-101029714 [+] | -1.9 |
| miR-1193 | chr14: 101030052-101030129 [+] | -1.0 |
| miR-543 | chr14: 101031987-101032064 [+] | -1.1 |
| miR-495 | chr14: 101033755-101033836 [+] | -1.9 |
|  |  |  |
| miR-181a-3p | chr1: 198859044-198859153 [-] | 1.4 |
| miR-181b-5p | chr1: 198858873-198858982 [-] | 1.4 |
| **miR-181b-3p** | chr1: 198858873-198858982 [-] | 3.8 |
|  |  |  |
| miR-30e-5p | chr1: 40754355-40754446 [+] | 1.8 |
| miR-30e-3p | chr1: 40754355-40754446 [+] | 1.5 |
| **miR-30c-1-3p** | chr1: 40757284-40757372 [+] | 3.3 |
|  |  |  |
| miR-100-5p | chr11: 122152229-122152308 [-] | 1.8 |
| **miR-100-3p** | chr11: 122152229-122152308 [-] | 4.8 |
| hsa-let-7a-2-3p | chr11: 122146522-122146593 [-] | 1.5 |
|  |  |  |
| miR-34b-5p | chr11: 111512938-111513021 [+] | -1.1 |
| miR-34b-3p | chr11: 111512938-111513021 [+] | -3.2 |
| miR-34c-5p | chr11: 111513439-111513515 [+] | -1.4 |
| **miR-34c-3p** | chr11: 111513439-111513515 [+] | -4.2 |
|  |  |  |
| **miR-331-5p** | chr12: 95308420-95308513 [+] | 3.0 |
| miR-3685 | chr12: 95309923-95309984 [+] | -1.0 |

^a^miRs in bold were found to be both differentially expressed by HPV16 E6/E7 and part of a miR cluster, unbolded miRs are also part of the miR clusters but did not meet threshold cutoffs; information regarding miR sequences has been removed for clarity
