# Supplementary material for: Modulation of microRNA-mRNA Target Pairs by Human Papillomavirus 16 Oncoproteins
Source: mBio. 2017 Jan 3;8(1):e02170-16. doi: 10.1128/mBio.02170-16 (PMC5210503; doi:10.1128/mBio.02170-16)
Supplement: TABLE S3 [file mbo006163134st3.docx]

**Table S3. Numbers and types of RNAs detected by RNAseq**

**(**threshold cut-offs: ≥ 10 reads; FDR ≤ 0.05; -2 ≥ FC ≥ 2)

| **RNA type** | **number** |
| --- | --- |
| protein coding RNA | 3,471 |
| pseudogene RNA | 2,703 |
| long, intervening non-coding RNA (lincRNA) | 1,452 |
| antisense RNA | 1,058 |
| long, non-coding transcripts in introns of coding genes w/no exon overlap (sense_intronic) | 193 |
| small nuclear RNA (snRNA) | 110 |
| RNA w/out ORF (processed_transcript) | 87 |
| small nucleolar RNA (snoRNA) | 70 |
| long, non-coding transcripts w/coding genes in introns on the same strand (sense_overlapping) | 35 |
| immunoglobulin (Ig) variable chain & T-cell receptor (TcR) RNA | 34 |
| inactivated immunoglobulin RNA | 28 |
| polymorphic pseudogene RNA | 16 |
