# Supplementary material for: Modulation of microRNA-mRNA Target Pairs by Human Papillomavirus 16 Oncoproteins
Source: mBio. 2017 Jan 3;8(1):e02170-16. doi: 10.1128/mBio.02170-16 (PMC5210503; doi:10.1128/mBio.02170-16)
Supplement: TABLE S5 [file mbo006163134st5.docx]

**Table S5. RNAs identified via the miR-mRNA pairing analysis to be potentially targeted by two or more miRs**

| **number of targeting miRs** | **number or name of RNAs potentially targeted** |
| --- | --- |
| 2 | 349 |
| 3 | 182 |
| 4 | 90 |
| 5 | 46 |
| 6 | 22 |
| 7 | ABCG4, ANKRD52, ATXN1, CCDC71L, CLCN5 ERBB3, FYCO1, KMT2C, MTF1, NFAT5 SH3PXD2A, SMURF1, SOX4, TANC2, ZNF365 |
| 8 | CFLAR, CREBRF, SHANK2 |
| 9 | CUX1, RORA |
| 10 | TRPS1 |
| 12 | ABL2 |
