# Supplementary material for: Modulation of microRNA-mRNA Target Pairs by Human Papillomavirus 16 Oncoproteins
Source: mBio. 2017 Jan 3;8(1):e02170-16. doi: 10.1128/mBio.02170-16 (PMC5210503; doi:10.1128/mBio.02170-16)
Supplement: TABLE S6 [file mbo006163134st6.docx]

**Table S6. Molecular and cellular functions identified via IPA core analysis associated with the RNAseq and potential miR target RNA data sets**

| **ALL RNAs** | |
| --- | --- |
| Molecular & Cellular Functions | p-value range^a^ |
| Cell Cycle | 5.46x10^-3^ – 3.13x10^-16^ |
| Cellular Assembly & Organization | 4.59x10^-3^ – 6.72x10^-14^ |
| DNA Replication, Recombination & Repair | 4.59x10^-3^ – 6.72x10^-14^ |
| Cell Morphology | 5.48x10^-3^ – 3.67x10^-7^ |
| Cellular Movement | 5.42x10^-3^ – 8.62x10^-6^ |
| **POTENTIAL miR TARGET RNAs** | |
| Molecular & Cellular Functions | p-value range^a^ |
| **Cellular Development^b^** | 1.60x10^-4^ – 3.15x10^-16^ |
| Cellular Movement | 1.53x10^-4^ – 7.49x10^-13^ |
| **Cellular Growth & Proliferation^b^** | 1.48x10^-4^ – 1.20x10^-11^ |
| **Molecular Transpor^b^** | 1.61x10^-4^ – 3.67x10^-11^ |
| Cell Morphology | 6.78x10^-5^ – 4.28x10^-10^ |

^a^The p-value for a given molecular or cellular function is calculated by comparing the number of genes in the data set that participate in that process to the total number of genes that are known to be associated with a given molecular or cellular function in the reference set. The p-value range refers to the range of p-values assigned to more specific molecular and cellular functions that fall within the larger molecular and cellular function categories shown.

^b^Bolded molecular and cellular functions are unique to the data set containing RNAs that are potential targets of miRs.
