# Supplementary material for: Modulation of microRNA-mRNA Target Pairs by Human Papillomavirus 16 Oncoproteins
Source: mBio. 2017 Jan 3;8(1):e02170-16. doi: 10.1128/mBio.02170-16 (PMC5210503; doi:10.1128/mBio.02170-16)
Supplement: TABLE S7 [file mbo006163134st7.docx]

**Table S7. Predicted upstream regulators identified via IPA core analysis based on gene expression changes observed in the RNAseq and potential miR target RNA data sets**

| **ALL RNAs** | | |
| --- | --- | --- |
| Upstream Regulator | P-value of Overlap^a^ | Predicted Activation |
| RABL6 | 1.02^-20^ | Activated |
| CCND1 | 1.59^-20^ | Activated |
| CDKN1A | 1.75^-19^ | Inhibited |
| EHF | 1.85^-19^ | Inhibited |
| dextran sulfate | 2.20^-17^ |  |
| **POTENTIAL miR TARGET RNAs** | | |
| Upstream Regulator | P-value of Overlap^a^ | Predicted Activation |
| TNF | 3.35^-17^ |  |
| TGFB1 | 3.92^-17^ | Inhibited |
| beta-estradiol | 1.68^-16^ |  |
| gefitinib | 6.27^-13^ |  |
| CREB1 | 1.27^-12^ |  |

^a^The overlap p‐value is calculated using Fisher’s Exact Test and determines whether there is a statistically significant overlap between genes in the dataset and genes that are regulated by a transcription factor. P‐values < 0.01 are considered significant and can be utilized to identify upstream regulators that may explain the observed gene expression changes.
