# Supplementary material for: Modulation of microRNA-mRNA Target Pairs by Human Papillomavirus 16 Oncoproteins
Source: mBio. 2017 Jan 3;8(1):e02170-16. doi: 10.1128/mBio.02170-16 (PMC5210503; doi:10.1128/mBio.02170-16)
Supplement: TABLE S8 [file mbo006163134st8.docx]

**Table S8. Comparison of trends in miR expression in HFKs expressing HPV16 E6/E7 with previously described HPV-associated miRs**

| **miR** | **Observed** | **Literature** | **References** | **Notes^a^** |
| --- | --- | --- | --- | --- |
| let-7a-5p | up | down | (1, 2) | CxCa |
| miR-10b-5p | up | up | (3) | CxCa, HNSCC |
| miR-100-5p | up | down | (4) | CxCa |
| miR-106b-5p | up | up | (5, 6) | OPC, VC |
| miR-124-3p | down | down | (7) |  |
| miR-1246 | down | down | (8) | CxCa |
| miR-1254 | up | down | (6) | VC |
| miR-125a-5p | up | down | (9) | CxCa |
| miR-125b-5p | up | down | (10) | VC |
| miR-1291 | down | down | (6) | VC |
| miR-139-5p | down | down | (3) | CxCa, HNSCC |
| miR-139-3p | down | down | (3) | CxCa, HNSCC |
| miR-143-3p | down | down | (11) | CxCa |
| miR-145-5p | down | down | (3, 12) | CxCa, HNSCC |
| miR-15a-5p | up | up | (3) | CxCa, HNSCC |
| miR-15b-5p | up | up | (13) | AC |
| miR-155-5p | up | up | (14) | OPC |
| miR-16-5p | up | up | (3, 15) | HNSCC, CxCa |
| miR-18a-5p | up | down | (14) | OPC |
| miR-186-5p | up | up | (6) | VC |
| miR-193a-5p | up | down | (6) | VC |
| miR-196a-5p | up | up | (16) | CxCa |
| miR-199a-5p | down | down | (3, 5) | CxCa, HNSCC, OPC |
| miR-199a-3p | up | down | (3) | CxCa, HNSCC |
| miR-199b-5p | down | down | (3) | CxCa, HNSCC |
| miR-20b-5p | up | up | (3, 17) | OPC, CxCa, HNSCC |
| miR-203a-3p | down | down | (18, 19) |  |
| miR-205-5p | down | down | (20) |  |
| miR-21-5p | up | up | (1, 2, 16, 21, 22) (6, 23) | CxCa, VC |
| miR-218-5p | up | down | (24-27) | CIN, OPC, PSCC |
| miR-22-3p | down | down | (6) | VC |
| miR-221-5p | up | up | (16, 28) | CxCa |
| miR-222-3p | up | up | (28) |  |
| miR-223-3p | down | down | (14) | OPC |
| miR-23b-3p | down | down | (29) |  |
| miR-24-3p | up | down | (20) |  |
| miR-25-3p | up | up | (5) | OPC |
| miR-26b-5p | up | down | (6) | VC |
| miR-27a-3p | up | up | (16) | CxCa |
| miR-29a-3p | up | down | (30) | CxCa |
| miR-29c-3p | up | up | (6) | VC |
| miR-31-5p | down | down | (14) | OPC |
| miR-3144-5p | down | down | (31) |  |
| miR-328 | down | down | (3) | CxCa, HNSCC |
| miR-34a-5p | down | down | (32-35) | CxCa |
| miR-375 | down | down | (36, 37) |  |
| miR-379-5p | down | down | (3) | CxCa, HNSCC |
| miR-381 | down | down | (3) | CxCa, HNSCC |
| miR-574-3p | down | down | (3) | CxCa, HNSCC |
| miR-875-5p | down | down | (31) |  |
| miR-9-5p | up | up | (5, 14, 17, 38) | CxCa, OPC |
| miR-9-3p | down | up | (17) | OPC |
| miR-93-5p | up | up | (5) | OPC |

^a^notes indicate when one or more literature references includes samples analyzed from HPV-associated cancer biopsies; AC= anal cancer; CxCa= cervical cancer; HNSCC= head and neck squamous cell carcinoma; OPC= oropharyngeal cancer; PSCC= penile squamous cell carcinoma; VC= vulvar cancer

1. **Shishodia G, Shukla S, Srivastava Y, Masaldan S, Mehta S, Bhambhani S, Sharma S, Mehrotra R, Das BC, Bharti AC.** 2015. Alterations in microRNAs miR-21 and let-7a correlate with aberrant STAT3 signaling and downstream effects during cervical carcinogenesis. Mol Cancer **14:**116.

2. **Shishodia G, Verma G, Srivastava Y, Mehrotra R, Das BC, Bharti AC.** 2014. Deregulation of microRNAs Let-7a and miR-21 mediate aberrant STAT3 signaling during human papillomavirus-induced cervical carcinogenesis: role of E6 oncoprotein. BMC Cancer **14:**996.

3. **Lajer CB, Garnaes E, Friis-Hansen L, Norrild B, Therkildsen MH, Glud M, Rossing M, Lajer H, Svane D, Skotte L, Specht L, Buchwald C, Nielsen FC.** 2012. The role of miRNAs in human papilloma virus (HPV)-associated cancers: bridging between HPV-related head and neck cancer and cervical cancer. Br J Cancer **106:**1526-1534.

4. **Li BH, Zhou JS, Ye F, Cheng XD, Zhou CY, Lu WG, Xie X.** 2011. Reduced miR-100 expression in cervical cancer and precursors and its carcinogenic effect through targeting PLK1 protein. Eur J Cancer **47:**2166-2174.

5. **Miller DL, Davis JW, Taylor KH, Johnson J, Shi Z, Williams R, Atasoy U, Lewis JS, Jr., Stack MS.** 2015. Identification of a human papillomavirus-associated oncogenic miRNA panel in human oropharyngeal squamous cell carcinoma validated by bioinformatics analysis of the Cancer Genome Atlas. Am J Pathol **185:**679-692.

6. **de Melo Maia B, Lavorato-Rocha AM, Rodrigues LS, Coutinho-Camillo CM, Baiocchi G, Stiepcich MM, Puga R, de ALL, Soares FA, Rocha RM.** 2013. microRNA portraits in human vulvar carcinoma. Cancer Prev Res (Phila) **6:**1231-1241.

7. **Wilting SM, van Boerdonk RA, Henken FE, Meijer CJ, Diosdado B, Meijer GA, le Sage C, Agami R, Snijders PJ, Steenbergen RD.** 2010. Methylation-mediated silencing and tumour suppressive function of hsa-miR-124 in cervical cancer. Mol Cancer **9:**167.

8. **Yang Y, Xie YJ, Xu Q, Chen JX, Shan NC, Zhang Y.** 2015. Down-regulation of miR-1246 in cervical cancer tissues and its clinical significance. Gynecol Oncol **138:**683-688.

9. **Fan Z, Cui H, Xu X, Lin Z, Zhang X, Kang L, Han B, Meng J, Yan Z, Yan X, Jiao S.** 2015. MiR-125a suppresses tumor growth, invasion and metastasis in cervical cancer by targeting STAT3. Oncotarget **6:**25266-25280.

10. **Nuovo GJ, Wu X, Volinia S, Yan F, di Leva G, Chin N, Nicol AF, Jiang J, Otterson G, Schmittgen TD, Croce C.** 2010. Strong inverse correlation between microRNA-125b and human papillomavirus DNA in productive infection. Diagn Mol Pathol **19:**135-143.

11. **Chen Y, Ma C, Zhang W, Chen Z, Ma L.** 2014. Down regulation of miR-143 is related with tumor size, lymph node metastasis and HPV16 infection in cervical squamous cancer. Diagn Pathol **9:**88.

12. **Gunasekharan V, Laimins LA.** 2013. Human papillomaviruses modulate microRNA 145 expression to directly control genome amplification. J Virol **87:**6037-6043.

13. **Myklebust MP, Bruland O, Fluge O, Skarstein A, Balteskard L, Dahl O.** 2011. MicroRNA-15b is induced with E2F-controlled genes in HPV-related cancer. Br J Cancer **105:**1719-1725.

14. **Gao G, Gay HA, Chernock RD, Zhang TR, Luo J, Thorstad WL, Lewis JS, Jr., Wang X.** 2013. A microRNA expression signature for the prognosis of oropharyngeal squamous cell carcinoma. Cancer **119:**72-80.

15. **Zheng ZM, Wang X.** 2011. Regulation of cellular miRNA expression by human papillomaviruses. Biochim Biophys Acta **1809:**668-677.

16. **Gocze K, Gombos K, Juhasz K, Kovacs K, Kajtar B, Benczik M, Gocze P, Patczai B, Arany I, Ember I.** 2013. Unique microRNA expression profiles in cervical cancer. Anticancer Res **33:**2561-2567.

17. **Hui AB, Lin A, Xu W, Waldron L, Perez-Ordonez B, Weinreb I, Shi W, Bruce J, Huang SH, O'Sullivan B, Waldron J, Gullane P, Irish JC, Chan K, Liu FF.** 2013. Potentially prognostic miRNAs in HPV-associated oropharyngeal carcinoma. Clin Cancer Res **19:**2154-2162.

18. **Melar-New M, Laimins LA.** 2010. Human papillomaviruses modulate expression of microRNA 203 upon epithelial differentiation to control levels of p63 proteins. J Virol **84:**5212-5221.

19. **McKenna DJ, McDade SS, Patel D, McCance DJ.** 2010. MicroRNA 203 expression in keratinocytes is dependent on regulation of p53 levels by E6. J Virol **84:**10644-10652.

20. **McKenna DJ, Patel D, McCance DJ.** 2014. miR-24 and miR-205 expression is dependent on HPV onco-protein expression in keratinocytes. Virology **448:**210-216.

21. **Liu S, Song L, Zhang L, Zeng S, Gao F.** 2015. miR-21 modulates resistance of HR-HPV positive cervical cancer cells to radiation through targeting LATS1. Biochem Biophys Res Commun **459:**679-685.

22. **Yao T, Lin Z.** 2012. MiR-21 is involved in cervical squamous cell tumorigenesis and regulates CCL20. Biochim Biophys Acta **1822:**248-260.

23. **Bumrungthai S, Ekalaksananan T, Evans MF, Chopjitt P, Tangsiriwatthana T, Patarapadungkit N, Kleebkaow P, Luanratanakorn S, Kongyingyoes B, Worawichawong S, Pientong C.** 2015. Up-Regulation of miR-21 Is Associated with Cervicitis and Human Papillomavirus Infection in Cervical Tissues. PLoS One **10:**e0127109.

24. **Wu DW, Chuang CY, Lin WL, Sung WW, Cheng YW, Lee H.** 2014. Paxillin promotes tumor progression and predicts survival and relapse in oral cavity squamous cell carcinoma by microRNA-218 targeting. Carcinogenesis **35:**1823-1829.

25. **Li Y, Liu J, Yuan C, Cui B, Zou X, Qiao Y.** 2010. High-risk human papillomavirus reduces the expression of microRNA-218 in women with cervical intraepithelial neoplasia. J Int Med Res **38:**1730-1736.

26. **Martinez I, Gardiner AS, Board KF, Monzon FA, Edwards RP, Khan SA.** 2008. Human papillomavirus type 16 reduces the expression of microRNA-218 in cervical carcinoma cells. Oncogene **27:**2575-2582.

27. **Barzon L, Cappellesso R, Peta E, Militello V, Sinigaglia A, Fassan M, Simonato F, Guzzardo V, Ventura L, Blandamura S, Gardiman M, Palu G, Fassina A.** 2014. Profiling of expression of human papillomavirus-related cancer miRNAs in penile squamous cell carcinomas. Am J Pathol **184:**3376-3383.

28. **Yang CJ, Shen WG, Liu CJ, Chen YW, Lu HH, Tsai MM, Lin SC.** 2011. miR-221 and miR-222 expression increased the growth and tumorigenesis of oral carcinoma cells. J Oral Pathol Med **40:**560-566.

29. **Au Yeung CL, Tsang TY, Yau PL, Kwok TT.** 2011. Human papillomavirus type 16 E6 induces cervical cancer cell migration through the p53/microRNA-23b/urokinase-type plasminogen activator pathway. Oncogene **30:**2401-2410.

30. **Li Y, Wang F, Xu J, Ye F, Shen Y, Zhou J, Lu W, Wan X, Ma D, Xie X.** 2011. Progressive miRNA expression profiles in cervical carcinogenesis and identification of HPV-related target genes for miR-29. J Pathol **224:**484-495.

31. **Lin L, Cai Q, Zhang X, Zhang H, Zhong Y, Xu C, Li Y.** 2015. Two less common human microRNAs miR-875 and miR-3144 target a conserved site of E6 oncogene in most high-risk human papillomavirus subtypes. Protein Cell **6:**575-588.

32. **Geng D, Song X, Ning F, Song Q, Yin H.** 2015. MiR-34a Inhibits Viability and Invasion of Human Papillomavirus-Positive Cervical Cancer Cells by Targeting E2F3 and Regulating Survivin. Int J Gynecol Cancer **25:**707-713.

33. **Wang X, Wang HK, McCoy JP, Banerjee NS, Rader JS, Broker TR, Meyers C, Chow LT, Zheng ZM.** 2009. Oncogenic HPV infection interrupts the expression of tumor-suppressive miR-34a through viral oncoprotein E6. RNA **15:**637-647.

34. **Wang X, Meyers C, Guo M, Zheng ZM.** 2011. Upregulation of p18Ink4c expression by oncogenic HPV E6 via p53-miR-34a pathway. Int J Cancer **129:**1362-1372.

35. **Li B, Hu Y, Ye F, Li Y, Lv W, Xie X.** 2010. Reduced miR-34a expression in normal cervical tissues and cervical lesions with high-risk human papillomavirus infection. Int J Gynecol Cancer **20:**597-604.

36. **Song L, Liu S, Zeng S, Zhang L, Li X.** 2015. miR-375 Modulates Radiosensitivity of HR-HPV-Positive Cervical Cancer Cells by Targeting UBE3A through the p53 Pathway. Med Sci Monit **21:**2210-2217.

37. **Bierkens M, Krijgsman O, Wilting SM, Bosch L, Jaspers A, Meijer GA, Meijer CJ, Snijders PJ, Ylstra B, Steenbergen RD.** 2013. Focal aberrations indicate EYA2 and hsa-miR-375 as oncogene and tumor suppressor in cervical carcinogenesis. Genes Chromosomes Cancer **52:**56-68.

38. **Liu W, Gao G, Hu X, Wang Y, Schwarz JK, Chen JJ, Grigsby PW, Wang X.** 2014. Activation of miR-9 by human papillomavirus in cervical cancer. Oncotarget **5:**11620-11630.
