# Supplementary material for: Modulation of microRNA-mRNA Target Pairs by Human Papillomavirus 16 Oncoproteins
Source: mBio. 2017 Jan 3;8(1):e02170-16. doi: 10.1128/mBio.02170-16 (PMC5210503; doi:10.1128/mBio.02170-16)
Supplement: TABLE S9 [file mbo006163134st9.docx]

**Table S9. Comparison of trends in miR expression in HFKs expressing HPV16 E6/E7 with study by Wang *et al.* 2014. PNAS, 111:4262-4267.**

| **miR** | **HPV16 E6E7/C (Observed)** | **HFK18/HFK (Literature)** | **Regulation^a^** | **Notes^b^** |
| --- | --- | --- | --- | --- |
| miR-16-5p | 3.0 | 3.2 | up | CaCx |
| miR-25-3p | 2.7 | 1.6 | up | CaCx |
| miR-92a-1-5p | 1.5 | 1.9 | up | CaCx |
| miR-93-5p | 1.9 | 2.3 | up |  |
| miR-106b-5p | 2.4 | 1.9 | up |  |
| miR-210 | -2.7 | 1.4 |  |  |
| miR-224-5p | -1.4 | 4.1 |  |  |
| miR-378a-5p | 2.7 | 1.9 | up | CaCx |
| miR-22-3p | -1.1 | −2.1 | down | CaCx |
| miR-24-3p | 1.4 | −1.5 |  |  |
| miR-27a-3p | 1.0 | −2.0 |  | CaCx |
| miR-29a-3p | 1.4 | −2.5 |  | CaCx |
| miR-100-5p | 1.8 | −2.8 |  | CaCx |

^a^shading indicates the trend in miR expression was not the same between our study and the compared study

^b^notes indicate when a miR was also detected in HPV-associated cervical cancer (CaCx) biopsies in the study by Wang *et al.* 2014. PNAS, 111:4262-4267
