# Supplementary material for: Modulation of microRNA-mRNA Target Pairs by Human Papillomavirus 16 Oncoproteins
Source: mBio. 2017 Jan 3;8(1):e02170-16. doi: 10.1128/mBio.02170-16 (PMC5210503; doi:10.1128/mBio.02170-16)
Supplement: TABLE S10 [file mbo006163134st10.docx]

**Table S10. Comparison of trends in miR expression in HFKs expressing HPV16 E6/E7 with study by Gunasekharan *et al.* 2013. J Virol, 87:6037-6043.**

| **miR** | **HPV16 E6E7/C (Observed)** | **viv-31gen/viv (Literature)** | **Regulation^a^** |
| --- | --- | --- | --- |
| miR-1246 | -1 | 31.7 |  |
| miR-335-3p | 14.5 | 10.6 | up |
| miR-1260b | -1.4 | 8.7 |  |
| miR-3613-5p | -1.4 | 5.9 |  |
| miR-1260a | -1 | 4.6 |  |
| miR-1285-3p | -1.9 | 4.1 |  |
| miR-576-5p | 2.8 | 4.0 | up |
| miR-615-5p | 3.7 | 3.9 | up |
| miR-92b-3p | 2.4 | 3.7 | up |
| miR-25-5p | 5.1 | 3.3 | up |
| miR-582-3p | 2.1 | -9.2 |  |
| miR-199a-5p | -1 | -8.7 | down |
| miR-214-3p | -1 | -8.4 | down |
| miR-143-3p | -2.3 | -7.3 | down |
| miR-145-5p | -1 | -7.1 | down |
| miR-145-3p | -1 | -6.9 | down |
| miR-369-3p | -1.3 | -6.5 | down |
| miR-655 | -1 | -6.4 | down |
| miR-493-3p | -1.1 | -6.2 | down |
| miR-493-5p | -1.1 | -5.3 | down |

^a^shading indicates the trend in miR expression was not the same between our study and the compared study
